# Sodium content targets for pre-packaged foods, China: a quantitative study and proposal

**DOI:** 10.2471/BLT.23.289992

**Published:** 2023-05-15

**Authors:** Puhong Zhang, Jiguo Zhang, Yuan Li, Le Dong, Feng J He, Mhairi Brown, Simone Pettigrew, Jacqui Webster, Rain Yamamoto, Chizuru Nishida, Aidong Liu, Xiaoguang Yang, Bing Zhang, Gangqiang Ding, Huijun Wang

**Affiliations:** a The George Institute for Global Health–China, Beijing, China.; b Key Laboratory of Trace Element Nutrition of the National Health Commission, National Institute for Nutrition and Health, Chinese Center for Disease Control and Prevention, 29 Nanwei Road, Beijing, 100050, China.; c Wolfson Institute of Population Health, Queen Mary University of London, London, England.; d The George Institute for Global Health, University of New South Wales, Sydney, Australia.; e Department of Nutrition and Food Safety, World Health Organization, Geneva, Switzerland.; f China National Center for Food Safety Risk Assessment, Beijing, China.

## Abstract

**Objective:**

To determine the contribution of pre-packaged foods to population sodium intake in China, and to propose sodium content targets for food subcategories used for the World Health Organization’s (WHO’s) global sodium benchmarks.

**Methods:**

The impact of four different approaches to reducing the sodium content of pre-packaged foods on population sodium intake was estimated using data from national databases covering the nutrient content and ingredients of 51 803 food products and food consumption by 15 670 Chinese adults. We recategorized food products using a food categorization framework developed for WHO’s global sodium benchmarks and adapted for China-specific foods.

**Findings:**

Pre-packaged foods, including condiments, contributed 1302.5 mg/day of sodium intake per adult in 2021, accounting for 30.1% of population sodium intake in China. Setting maximum sodium content levels using a 90th-percentile target would reduce sodium intake from pre-packaged foods by 96.2 mg/day, corresponding to a 1.9% reduction in population intake. Using the 75th-percentile, a fixed 20% reduction and WHO benchmark targets would further reduce intake by 262.0 mg/day (5.2% population intake), 302.8 mg/day (6.0% population intake) and 701.2 mg/day per person (13.9% population intake), respectively. Maximum sodium content levels based on revised 20% reduction targets were proposed because they should result in substantial and acceptable reductions in sodium content for most food subcategories: overall sodium intake would decline by 305.0 mg/day per person, and population intake by 6.1%.

**Conclusion:**

This study provides the scientific rationale for government policy on setting targets for food sodium content in China. Simultaneous action on discretionary salt use should also be taken.

## Résumé

**Objectif:**

Déterminer dans quelle mesure les denrées alimentaires préemballées contribuent à l'apport en sodium au sein de la population chinoise et proposer des objectifs pour en limiter la teneur parmi les sous-catégories d'aliments reprises dans les critères de référence mondiaux de l'Organisation mondiale de la Santé (OMS).

**Méthodes:**

Nous avons estimé l'impact de quatre approches différentes visant à réduire la teneur en sodium des denrées alimentaires préemballées sur l'apport sodé de la population en utilisant les informations issues de bases de données nationales sur la teneur en nutriments et les ingrédients de 51 803 produits alimentaires ainsi que la consommation en nourriture de 15 670 adultes chinois. Nous avons reclassé les produits alimentaires à l'aide d'un système de catégorisation des aliments développé dans le cadre des critères de référence mondiaux de l'OMS et adapté aux habitudes alimentaires du pays.

**Résultats:**

Les denrées alimentaires préemballées, y compris les condiments, représentaient un apport sodé de 1302,5 mg/jour par adulte en 2021, ce qui équivaut à 30,1% de l'apport en sodium au sein de la population chinoise. Établir un objectif fondé sur le 90^e^ percentile afin de définir une teneur maximale en sodium permettrait de réduire l'apport sodé des denrées alimentaires préemballées de 96,2 mg/jour, ce qui correspond à une diminution de 1,9% de l'apport dans la population. Utiliser le 75^e^ percentile, une réduction fixe de 20% et les critères de référence mondiaux de l'OMS ferait encore baisser l'apport de 262,0 mg/jour (5,2% de l'apport dans la population), 302,8 mg/jour (6,0% de l'apport dans la population) et 701,2 mg/jour par personne (13,9% de l'apport dans la population) respectivement. Enfin, une teneur maximale en sodium inspirée des nouveaux objectifs de 20% de réduction a été proposée car elle devrait entraîner un recul considérable et acceptable de la teneur en sodium pour la plupart des sous-catégories d'aliments: l'apport sodé global diminuerait de 305,0 mg/jour par personne et l'apport dans la population de 6,1%.

**Conclusion:**

Cette étude fournit les arguments scientifiques nécessaires à la mise en œuvre d'une politique gouvernementale qui définirait des objectifs relatifs à la teneur en sodium des denrées alimentaires en Chine. Des actions concomitantes en matière d'utilisation du sel de table et de cuisine devraient également être entreprises.

## Resumen

**Objetivo:**

Determinar cómo los alimentos preenvasados propician la ingesta de sodio en la población china, y proponer objetivos centrados en el contenido de sodio de las subcategorías alimenticias utilizadas en las referencias mundiales de sodio de la Organización Mundial de la Salud (OMS).

**Métodos:**

Se evaluó la repercusión de cuatro enfoques diferentes destinados a reducir el contenido de sodio de los alimentos preenvasados en la ingesta de sodio poblacional. Para ello, se utilizó información de las bases de datos nacionales acerca del contenido en nutrientes y los ingredientes de 51 803 productos alimenticios, así como sobre el consumo de alimentos de 15 670 chinos adultos. Restablecimos las categorías de los productos alimenticios utilizando un esquema de clasificación alimenticia desarrollado para englobar las referencias mundiales de sodio de la OMS, adaptado a alimentos específicos de China.

**Resultados:**

Los alimentos preenvasados, incluidos los condimentos, representaban 1302,5 mg/día de la ingesta de sodio por adulto en 2021, lo que suponía un 30,1% de la ingesta de sodio de la población china. Establecer niveles máximos de contenido en sodio utilizando como objetivo un percentil 90 reduciría la ingesta de sodio de los alimentos preenvasados en 96,2 mg/día, lo que corresponde a una reducción del 1,9% en la ingesta poblacional. Utilizando el percentil 75, una reducción fija del 20% y los objetivos de referencia de la OMS se seguiría reduciendo la ingesta en 262,0 mg/día (5,2% de la ingesta poblacional), 302,8 mg/día (6,0% de la ingesta poblacional) y 701,2 mg/día por persona (13,9% de la ingesta poblacional) respectivamente. Se propusieron niveles máximos de contenido en sodio basados en objetivos revisados relativos a una reducción del 20%, lo que supondría una disminución significativa y adecuada del contenido de sodio de la mayoría de subcategorías alimenticias: la ingesta global de sodio disminuiría en 305,0 mg/día por persona, y la ingesta poblacional se reduciría en un 6,1%.

**Conclusión:**

El presente estudio ofrece fundamentos científicos relevantes para la política gubernamental, acerca del establecimiento de objetivos centrados en el contenido de sodio alimenticio de productos de China. Asimismo, se deberían adoptar medidas simultáneas relativas al consumo de sal opcional.

## ملخص

**الغرض:**

تحديد مساهمة الأطعمة سابقة التعبئة في استهلاك السكان من الصوديوم في الصين، واقتراح المحتوى المستهدف من الصوديوم في الفئات الفرعية من الأغذية المستخدمة لأغراض معايير الصوديوم العالمية لدى منظمة الصحة العالمية (WHO).

**الطريقة:**

تم تقدير تأثير أربعة أساليب مختلفة لتقليل محتوى الصوديوم في الأطعمة سابقة التعبئة، على استهلاك السكان من الصوديوم باستخدام بيانات من قواعد البيانات الوطنية التي تغطي المحتوى الغذائية، ومكوناتها في 51803 منتجًا غذائيًا، واستهلاك الغذاء بواسطة 15670 بالغًا صينيًا. قمنا بإعادة تصنيف المنتجات الغذائية باستخدام إطار عمل تصنيف الأغذية الذي تم تطويره لأغراض معايير الصوديوم العالمية لدى منظمة الصحة العالمية، والتي تم تكييفه مع الأطعمة الخاصة بالصين.

**النتائج:**

ساهمت الأطعمة سابقة التعبئة، بما في ذلك التوابل، في 1302.5 ملج/يوم من استهلاك الصوديوم لكل شخص بالغ في عام 2021، وهو ما يمثل %30.1 من استهلاك السكان للصوديوم في الصين. إن تحديد مستويات الحد الأقصى من محتوى الصوديوم باستخدام مستهدف النسبة المئوية التسعينية، من شأنه أن يقلل من استهلاك الصوديوم من الأطعمة سابقة التعبئة بنسبة 96.2 ملج/يوم، وهو ما يمثل انخفاضًا بنسبة %1.9 في استهلاك السكان. باستخدام النسبة المئوية الخامسة والسبعين، فإن التقليل الثابت بنسبة %20 والأهداف المعيارية لمنظمة الصحة العالمية من شأنها أن تقلل بشكل أكبر من الاستهلاك بمقدار 262.0 ملج/يوم (%5.2 من استهلاك السكان)، و302.8 ملج/يوم (%6.0 من استهلاك السكان)، و701.2 ملج/يوم للشخص الواحد (%13.9 من استهلاك السكان)، على التوالي. تم اقتراح مستويات الحد الأقصى من محتوى الصوديوم بناءً على مستهدفات خفض منقحة بنسبة %20 لأنه يجب أن تؤدي إلى تخفيضات كبيرة ومقبولة في محتوى الصوديوم لمعظم الفئات الفرعية للأغذية: سينخفض استهلاك الصوديوم الكلي بمقدار 305.0 ملج/يوم لاستهلاك الشخص والسكان، بنسبة %6.1.

**الاستنتاج:**

تقدم هذه الدراسة الأساس المنطقي العلمي لسياسة الحكومة بشأن تحديد مستهدفات لمحتوى الصوديوم في الغذاء في الصين. يجب أيضًا اتخاذ إجراء متزامن بشأن الاستخدام التقديري للملح.

## 摘要

**目的:**

旨在确定预包装食品对中国全人群钠摄入量的影响，并基于世界卫生组织 (WHO) 制定全球钠含量基准时所采用的食品分类体系提出最高钠含量标准的建议。

**方法:**

使用全国 51,803 种食品营养成分及 15,670 名中国成年人食品消费量的数据，评估四种不同的降低预包装食品钠含量的方法对人群钠摄入量的影响。我们使用为世界卫生组织全球钠含量基准制定的食品分类框架对食品进行了重新分类，并针对中国特有的食品进行了调整。

**结果:**

2021 年，成年人预包装食品（包括调味品）的人均钠摄入量为 1302.5 毫克/ 天，占中国人群钠摄入量的 30.1%。使用第 90 百分位数设定最高钠含量水平将使通过预包装食品的钠摄入量减少 96.2 毫克 / 天，相当于使人群钠摄入量减少 1.9%。使用第 75 百分位、固定减少 20% 的摄入量和世卫组织钠基准为最高标准，将分别使钠摄入量减少 262.0 毫克 / 天（人群摄入量 5.2%）、302.8 毫克 / 天（人群摄入量 6.0%）和 701.2 毫克 / 天（人群摄入量 13.9%）。本文建议采用修订后的固定减少 20% 摄入量的标准作为最高钠含量水平，因为它们将使大多数食品亚类的钠含量在可接受的范围内大幅减少 ：人均钠摄入量将减少 305.0 毫克 / 天，相当于人群钠摄入量减

**结论:**

本研究为中国政府制定食品最高钠含量标准提供了科学依据。同时也建议采取措施，同步控制烹调用盐的使用。本研究为中国政府制定食品钠含量指标提供了科学依据。同时也应采取行动控制止随意使用食盐。

## Резюме

**Цель:**

Определить степень влияния фасованных продуктов питания на потребление натрия населением Китая и предложить целевые показатели содержания натрия в подкатегориях продуктов питания, глобально используемых Всемирной организацией здравоохранения (ВОЗ) для определения потребления натрия.

**Методы:**

Влияние четырех различных подходов к снижению содержания натрия в фасованных продуктах питания на потребление натрия населением было оценено с использованием данных из национальных баз данных, включающих информацию о содержании питательных веществ и ингредиентов в 51 803 продуктах питания и сведения о потреблении пищи 15 670 взрослыми жителями Китая. Продукты питания были переклассифицированы с использованием системы классификации, разработанной для глобальных эталонов ВОЗ по содержанию натрия и адаптированной для продуктов питания, специфичных для Китая.

**Результаты:**

В 2021 году на долю предварительно расфасованных продуктов питания, включая приправы, приходилось 1302,5 мг/день потребления натрия на взрослого человека, что составляло 30,1% потребления натрия населением Китая. Установление максимальных уровней содержания натрия с использованием целевого показателя 90-го процентиля позволит снизить потребление натрия из фасованных продуктов питания на 96,2 мг/день, что соответствует сокращению потребления населением на 1,9%. Если использовать в качестве целевого показателя 75-й процентиль, фиксированное 20%-е снижение или достижение контрольных показателей ВОЗ, потребление снизится на 262,0 мг/день (потребление населением снизится на 5,2%), 302,8 мг/день (потребление населением – на 6,0%) и 701,2 мг/день на человека (потребление населением – на 13,9%) соответственно. Было предложено установить максимальные уровни содержания натрия на основе пересмотренных целевых показателей снижения на 20%, поскольку они должны привести к существенному и приемлемому снижению содержания натрия в большинстве подкатегорий продуктов питания: общее потребление натрия снизится на 305,0 мг/день на человека, а потребление населением – на 6,1%.

**Вывод:**

Данное исследование дает научное обоснование государственной политики по установлению целевых показателей содержания натрия в продуктах питания в Китае. Одновременно следует принять меры по разумному потреблению соли.

## Introduction

Sodium is an essential nutrient for which humans have a physiological need of as little as 200 to 500 mg/day.^1^^,^^2^ The primary dietary contributor is the salt (i.e. sodium chloride) added during cooking or at the table, and hidden in processed foods. One gram of sodium is equivalent to 2.5 g of salt. Other sodium compounds, such as sodium glutamate, and some other food additives may also contribute to sodium intake. 

Excessive sodium intake is associated with an increase in blood pressure and the risk of cardiovascular diseases and other chronic conditions.^3^^–^^6^ Global burden of disease studies showed that excessive sodium intake led to 1.7 million deaths in China and 3.2 million deaths globally in 2017.^7^^,^^8^ The World Health Organization (WHO) strongly recommends a sodium intake for adults of less than 2 g/day (i.e. 5 g/day of salt).^9^ However, the current global average salt intake is estimated to be 10.78 g/day per person, which is more than double WHO’s recommendation and far exceeds physiological requirements.^10^

In many developed countries, pre-packaged food accounts for more than three quarters of population sodium intake.^11^ Consequently, one of the best strategies for reducing sodium intake has been to harness the food industry to reformulate products by gradually lowering targets for sodium content.^12^ WHO reports that 65 countries around the world, mostly high- or middle-income countries, have implemented this policy.^10^

In 2021, WHO established global sodium benchmarks for 58 subcategories of food that were based on the lowest feasible maximum value for each subcategory, as defined in existing national or regional targets.^13^ These benchmarks provide a clear reference point for countries wishing to set or develop maximum sodium levels for foods. However, strategies for gradually reducing the sodium content of foods must avoid sharp reductions and the associated taste shock for consumers, especially in countries like China where dietary sodium primarily comes from cooking or table salt, and where consumers’ taste preferences are largely influenced by foods prepared at home and in restaurants.^11^^,^^14^^–^^17^

Although the recommended sodium intake for individuals in China is the same as WHO’s recommendation, the national goal set in 2016 was to reduce the population sodium intake by 20% by 2030,^18^ which is more conservative than WHO’s comparable global target of a 30% reduction by 2025.^13^ As around 80% of sodium intake in China comes from salt and condiments added during cooking,^19^^,^^20^ the government has been implementing several national salt reduction programmes since the 1990s that target catering and family cooks.^21^ As a result, average household salt use decreased from 10.4 g/day per person in 2010 to 2012 to 9.3 g/day per person in 2015,^22^ still above recommended level.

Nevertheless, despite the continuing increase in the sale of pre-packaged food in China and the relatively high sodium content of pre-packaged food,^23^^–^^27^ efforts to reduce sodium content by promoting food reformulation in the country have been very limited. Until now, except for policy on the mandatory labelling of the sodium content of pre-packaged foods, no official regulation has been issued to incentivize industry to reduce salt use. There exists only the set of voluntary sodium targets included in the Guideline for Salt Reduction for the Chinese Food Industry, which was issued in 2019.^28^ The Guideline sets mean and maximum levels for the sodium content of individual food categories in China's unique food categorization framework, but sodium target levels were derived using data on only 9000 food products.

With the aim of accelerating the reduction in population sodium intake in China by reformulating pre-packaged foods, we explored the contribution of these foods to sodium intake, and assessed the potential impact of different approaches to reducing the sodium content of foods on population intake. In addition, we propose a set of maximum sodium target levels for different food subcategories to inform government policy on target setting.

## Methods

The study involved data from two national databases covering the nutrient content and ingredients of pre-packaged foods and food consumption by Chinese adults, respectively. This study was approved by the Institutional Review Board of the National Institute for Nutrition and Health, Chinese Center for Disease Control and Prevention (no. 2018–005).

The Chinese pre-packaged food database contains information on the nutrient content and ingredients of pre-packaged foods available for sale on the Chinese market.^29^ Data collection started in March 2017 using Shixianzhi, a local FoodSwitch app (George Institute for Global Health, Newtown, Australia) for WeChat (Tencent Holdings Ltd, Shenzhen, China).^25^^,^^30^ Between 2017 and 2018, data on 32 000 products were collected from the top 10 supermarkets in two provincial capitals in north and south China, respectively. Since January 2019, the food database has constantly been supplemented by consumers nationwide using crowdsourcing.^29^^,^^31^ During crowdsourcing, data were collected only on products that did not already exist in the Shixianzhi database to avoid duplication. For this study, we examined data on 76 354 products collected between March 2017 and February 2021. We excluded products that contained almost no sodium, pure sodium compounds, baby foods and products whose sodium content was unknown.

Food consumption data were obtained from the China Health and Nutrition Survey,^32^ an ongoing longitudinal household-based survey initiated in 1989 and conducted every 2 to 4 years, that was established to study the effect of socioeconomic change on nutrition and health. Households across different geographical regions of China with varying levels of economic development were selected using a multistage, random-cluster, sampling strategy.^32^ In each survey, trained interviewers interviewed all family members. Information on the consumption of individual foods was collected using three consecutive 24-hour dietary recalls on two weekdays and one weekend day, respectively. The name and amount of each food consumed, including pre-packaged foods, were recorded.^33^ For our study, we used data on pre-packaged food consumption collected in 2018 from all 15 670 adult survey participants in 15 provinces.

Initially, food products were categorized using a hierarchical category tree developed by the Global Food Monitoring Group.^34^ However, for our study, we recategorized all products in line with the food categorization framework used for WHO’s global sodium benchmarks.^13^ During this process, we excluded the existing category 5 (edible ices) because there was no product in this category on the Chinese market, and we added a new category 19 (egg and egg products) because there were hundreds of egg products with a relatively high sodium content in China. In addition, we added some new subcategories and redefined or expanded the descriptions of some subcategories to take into account China-specific food products. Details are available from the online repository.^35^ The original WHO category and subcategory names were unchanged. Our analysis and results are based on this adapted categorization.

### Statistical analysis

All sodium content values were converted into mg/100 g of food product. For each product subcategory, sodium content is described using the mean, standard deviation (SD), standardized SD (i.e. the SD divided by the mean), minimum, maximum and the 25th, 50th (i.e. median), 75th and 90th percentiles.

The population sodium intake attributable to pre-packaged foods is the sum of the sodium intake of all individual food subcategories and is expressed in mg/day per person. Sodium intake from each subcategory was calculated as the median sodium content of foods in the subcategory (in mg/100 g) multiplied by their consumption (in g/day per person) and divided by 100, where consumption of food in a subcategory (in g/day per person) was the total amount of food in the subcategory consumed over three days by all survey participants (in g) divided by the total number of participants and divided by 3.

Following the approach used to establish WHO’s sodium benchmarks, we decided to set individual maximum sodium target levels for each food subcategory. We considered three different types of targets in exploring suitable maximum sodium food content levels: (i) percentile targets; (ii) fixed percentage reduction targets; and (iii) WHO benchmarks. With the 75th percentile target, the 25% of food products in each subcategory that had a sodium content above the 75th percentile for that subcategory were reformulated to ensure their sodium content did not exceed the existing 75th percentile. With the 90th percentile target, an analogous 10% of food products in each subcategory were reformulated to ensure their sodium content did not exceed the 90th percentile. For a fixed percentage reduction target, the goal was to achieve a fixed percentage reduction in mean sodium content from baseline in each subcategory. With this approach, the projected maximum sodium content of individual food products in a subcategory was reduced reiteratively in steps of 1 mg/100 g until the desired percentage reduction in the mean sodium content of the whole subcategory was achieved. In each step, only products with a sodium content above the maximum for that step would have to be reformulated. In our study, we considered only a 20% reduction in sodium content, which reflects the national goal of a 20% reduction in sodium intake by 2030.

Several indicators were used to reflect the impact of different maximum sodium target levels on the number of foods to be reformulated and on sodium intake, and to guide the selection of the most suitable targets. It was assumed that target levels had been achieved and that the sodium content of products would not change once these targets had been achieved. The indicators were: (i) the proportion of products in each food subcategory that would have to be reformulated (i.e. the proportion of products with a sodium content above the subcategory target); (ii) the resulting reduction in mean sodium content for each subcategory (i.e. the difference in the mean sodium content of foods in the subcategory, before and after reformulation, in mg/100 g); (iii) the resulting reduction in population sodium intake from a subcategory, in mg/day per person, calculated as the reduction in sodium content for the subcategory (in mg/100 g) multiplied by the amount consumed from the subcategory (in g/day per person) and divided by 100; and (iv) the resulting reduction in sodium intake from all pre-packaged foods (i.e. the sum of the individual reductions across all subcategories). 

The impact of different target levels could be seriously affected by the distribution of, or variation in, sodium content across foods in a subcategory. Consequently, to help identify the most suitable targets, we drew scatter plots to visualize the relationship between: (i) the reduction in mean sodium content achieved in a subcategory if targets were met, and the standardized SD in sodium content for that subcategory; and (ii) the proportion of products in a subcategory that would have to be reformulated if targets were met, and the standardized SD in sodium content for that subcategory.

### Selection of maximum targets

Our recommended sodium target levels were based on two criteria. First, the targets should result in a relative reduction in sodium intake from all pre-packaged foods consistent with the national 20% reduction goal, while bearing in mind that discretionary salt use, which dominates consumption in China, must be similarly reduced in parallel. Second, the targets should result in a substantial and acceptable reduction in sodium content for each food subcategory, especially for the main sodium contributors, and consider: (i) the effect of the sodium content reduction on consumers’ changing taste for salt; and (ii) the challenges posed to food producers by the number of foods that have to be reformulated.

## Results

**Fig. 1:**
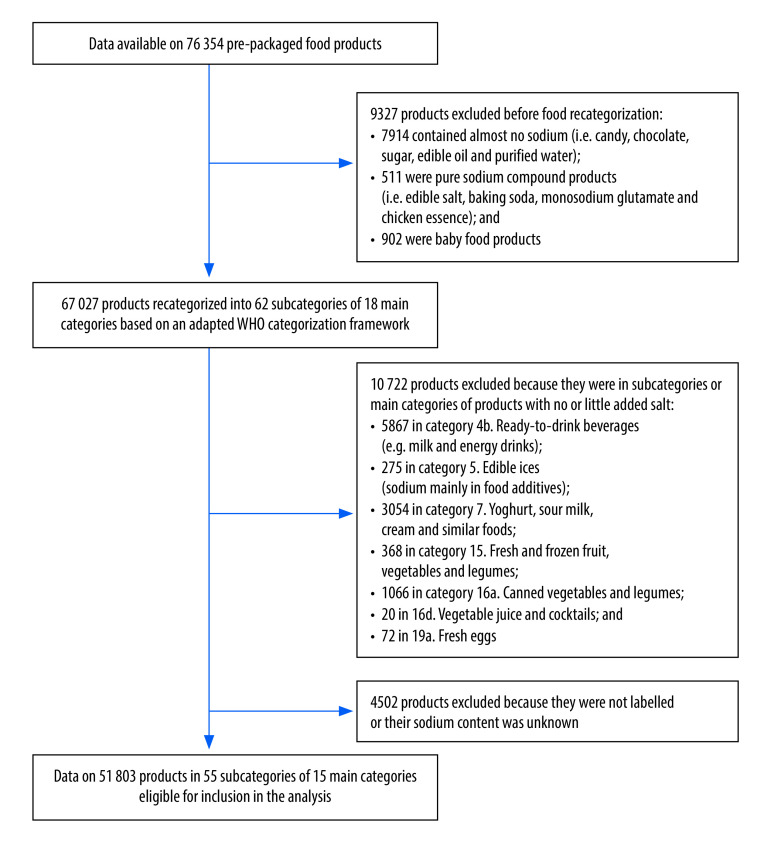
Selection of food products for sodium reduction analysis, China, 2021

Of 76 354 pre-packaged food products in the Chinese database, 67 027 (87.8%) were recategorized into 62 subcategories of 18 main categories based on our adapted WHO categorization framework, and 51 803 (67.9%) in 55 subcategories of 15 main categories were eligible for inclusion in the analysis (Fig. 1). The 2018 China Health and Nutrition Survey reported on 3020 products in 51 subcategories consumed by 15 670 adults. Details of consumption for each food subcategory are available from the online repository.^35^

WHO: World Health Organization.

Note: Food subcategories were derived from the World Health Organization’s food categorization framework,^13^ with some adaptation to take China-specific food products into account.

### Sodium content of foods

The sodium content of different Chinese food product subcategories is listed in Table 1 and is described in detail in the online repository.^35^ Mean sodium content ranged from 93 mg/100 g to 7129 mg/100 g and the standardized SD ranged from 0.38 to 2.82. Sodium intake per adult from pre-packaged foods based on the median sodium content of individual subcategories was 1302.5 mg/day (i.e. 3.3 g of salt; Table 2); this accounted for 30.1% of population salt intake in China, which was 11 g/day per person in 2020.^36^ The subcategory of soy sauce and fish sauce contributed most to sodium intake (median: 616.6 mg/day per person, which accounted for 47.3% of the 1302.5 mg/day from all pre-packaged foods), followed by the categories of ready-to-eat meals, and of salted butter, butter blends, margarine and oil-based spreads. The top six and top 12 subcategories accounted for 83.4% and 95.1% of sodium intake from all pre-packaged foods, respectively (Table 2).

**Table 1:** Sodium content of pre-packaged foods, by subcategory, China, 2021

Food product subcategory classification and description^a,b^ (*n* = 55)	No. food products in subcategory	Sodium content of food products^c^ (mg/ 100 g)^d^			
		Mean (SD)	Standardized SD^e^	Median (IQR)	Minimum–Maximum
18aii. Bouillon and soup stock (concentrated)	217	7 129 (6 776)	0.95	5 682 (2 956–8 327)	10–53 000
9gii. Soups (dry soup only; concentrated)	221	5 261 (4 605)	0.88	4 624 (1 450–8 330)	6–25 606
18f. Soy sauce and fish sauce	1898	5 213 (2 436)	0.47	5 195 (3 600–6 830)	10–17 692
18h. Marinades and thick pastes	2018	4 115 (5 746)	1.40	2 418 (403–5 118)	10–77 726
18e. Condiments	785	2 855 (2 145)	0.75	2 553 (1 527–3 780)	3–30 813
14c. Processed fish and seafood products, non-heat-treated	76	2 581 (1 737)	0.67	1 710 (1 234–4 892)	25–5 490
18g. Other Asian-style sauces	22	2 262 (1976)	0.87	1 914 (605–3 758)	72–8 200
16b. Pickled vegetables	1799	1 903 (1 595)	0.84	1 770 (1 064–2 292)	7–26 891
17b. Meat analogues	44	1 706 (1 136)	0.67	1 534 (786–2 412)	154–6 500
9bii. Pasta, noodles and rice or grains with sauce or seasoned (dry mix, concentrated)	2448	1 646 (2 171)	1.32	1 347 (482–2 130)	2–32 024
14h. Comminuted meat products, non-heat preservation	124	1 566 (954)	0.61	1 440 (791–2 211)	37–5 910
14i. Processed fish and seafood, heat-treated (cooked)	1231	1 413 (1 050)	0.74	1 337 (854–1 694)	9–18 550
14f. Whole muscle meat products, non-heat preservation	1551	1 411 (537)	0.38	1 460 (1 047–1 708)	18–9 447
19b. Processed eggs and egg products	280	1 160 (1 361)	1.17	816 (621–1 396)	125–20 800
18b. Cooking sauces including pasta sauces and tomato sauces (not concentrated)	717	1 151 (1 869)	1.62	546 (325–981)	6–22 200
14g. Comminuted meat products, heat-treated (cooked)	2759	1 131 (575)	0.51	1 037 (834–1 349)	17–14 519
14eii. Whole muscle meat products, heat-treated (refrigerated products)	65	962 (602)	0.63	886 (420–1 370)	6–2 960
18d. Emulsion-based dips, sauces and dressings	196	950 (758)	0.80	760 (600–1 196)	10–9 030
17a. Tofu and tempeh	911	936 (612)	0.65	929 (636–1 212)	3–6 573
9f. Ready-to-eat meals composed of a combination of carbohydrate and either vegetable or meat, or all three combined	1287	873 (987)	1.13	463 (120–1 232)	4–5 683
14ei. Whole muscle meat products, heat-treated (frozen and canned products)	264	818 (486)	0.59	750 (598–932)	30–3 530
14a. Canned fish	418	791 (551)	0.70	660 (431–976)	62–5 200
14b. Processed fish and seafood products, raw	425	783 (1 542)	1.97	417 (192–690)	1–17 706
3d. Extruded snacks	2968	773 (737)	0.95	570 (311–874)	6–5 683
8a. Fresh unripened cheese	14	721 (570)	0.79	550 (291–952)	117–1 969
16c. Olives and sundried tomatoes	4083	714 (1 837)	2.57	91 (22–519)	1–27 780
3c. Potato, vegetable and grain chips	1988	690 (523)	0.76	593 (399–838)	6–9 702
14d. Raw meat products and preparations	443	639 (899)	1.41	536 (316–735)	5–10 320
8c. Semi-hard ripened cheese	196	636 (491)	0.77	600 (320–745)	10–3 500
16g. Battered or breaded vegetables	43	590 (1 266)	2.15	258 (111–570)	10–8 000
8g. Processed cheese	126	583 (413)	0.71	471 (243–900)	30–1 903
9gi. Soups (ready-to-serve, canned and refrigerated soups)	14	553 (453)	0.82	356 (263–507)	232–1 660
3a. Crackers and savoury biscuits	955	490 (285)	0.58	480 (294–634)	3–2 943
9c. Pizza and pizza snacks	58	471 (222)	0.47	410 (336–544)	72–1 220
9d. Sandwiches and wraps	79	457 (284)	0.62	388 (260–616)	20–1 398
3b. Nuts, seeds and kernels	3368	414 (559)	1.35	298 (86–574)	1–7 156
10a. Salted butter, butter blends, margarine and oil-based spreads	59	405 (328)	0.81	480 (20–695)	6–1 000
12. Fresh or dried pasta, noodles, rice and grains	2680	398 (653)	1.64	192 (14–650)	1–20 100
18c. Dips and dipping sauces	331	340 (958)	2.82	23 (10–62)	2–5 048
11c. Flatbreads	117	321 (223)	0.69	304 (179–411)	6–1 417
1b. Nut butters	46	313 (169)	0.54	334 (196–488)	9–536
2e. Pancakes, waffles and French toast	68	292 (135)	0.46	292 (200–363)	70–970
11b. Leavened bread	370	282 (163)	0.58	255 (193–361)	2–1 640
2g. Dry mixes for making cakes, sweet biscuits, pastries and other sweet bakery wares	62	268 (169)	0.63	238 (182–305)	29–879
2a. Cookies and sweet biscuits	5191	261 (174)	0.67	230 (140–347)	6–1 382
11a. Sweet and raisin breads	817	240 (113)	0.47	222 (180–277)	9–1 404
4a. Solids and powders	3286	226 (542)	2.40	145 (50–250)	1–9 810
1a. Granola and cereal-type bars	156	201 (137)	0.68	180 (101–276)	5–930
2b. Cakes and sponges	1743	201 (179)	0.89	180 (98–271)	2–3 110
2c. Pies and pastries	1110	194 (268)	1.38	132 (57–235)	2–2 969
9bi. Pasta, noodles and rice or grains with sauce or seasoned (prepared)	167	193 (404)	2.09	37 (25–85)	1–2 361
6b. Highly processed breakfast cereals	1122	175 (242)	1.38	100 (30–236)	2–2 300
9e. Prepared salads	5	154 (65)	0.42	140 (125–174)	79–253
2d. Baked and cooked desserts	92	120 (162)	1.34	68 (41–110)	6–885
6a. Minimally processed breakfast cereals (includes all types – prepared, ready-made and dry mixes)	290	93 (146)	1.57	20 (8–146)	2–1 080

IQR: interquartile range; SD: standard deviation.

^a^ Subcategories are ordered from high to low according to their mean sodium content.

^b^ Food subcategories were based on the World Health Organization’s food categorization framework,^13^ with some adaptation to take China-specific food products into account.

^c^ The sodium content of food products was derived from the FoodSwitch database.^31^

^d^ All figures are in mg/100 g except for the standardized SD

^e^ The standardized SD is the SD divided by the mean.

**Table 2:** Contribution of pre-packaged foods to population sodium intake, by subcategory, China, 2021

Food product subcategory classification and description^a,b,c^ (*n* = 43)	Parameters for food products in subcategory			
	Median sodium content,^d^ mg/100 g	Consumption,^e^ g/day per person	Sodium intake,^f^ mg/day per person	% of total sodium intake
18f. Soy sauce and fish sauce	5195	11.87	616.60	47.34
9f. Ready-to-eat meals composed of a combination of carbohydrate and either vegetable or meat, or all three combined	463	31.11	144.10	11.06
10a. Salted butter, butter blends, margarine, and oil-based spreads	480	19.13	91.80	7.05
9bii. Pasta, noodles and rice or grains with sauce or seasoned (dry mix, concentrated)	1347	6.41	86.30	6.62
16b. Pickled vegetables	1770	4.81	85.20	6.54
14f. Whole muscle meat products, non-heat preservation	1460	4.29	62.60	4.81
17a. Tofu and tempeh	929	4.36	40.50	3.11
18b. Cooking sauces including pasta sauces and tomato sauces (not concentrated)	546	5.52	30.10	2.31
18e. Condiments	2553	0.89	22.60	1.74
14g. Comminuted meat products, heat-treated (cooked)	1037	2.12	21.90	1.68
14h. Comminuted meat products, non-heat preservation	1440	1.38	19.90	1.53
18h. Marinades and thick pastes	2418	0.71	17.20	1.32
11b. Leavened bread	255	4.89	12.50	0.96
3a. Crackers and savoury biscuits	480	2.09	10.00	0.77
14i. Processed fish and seafood, heat-treated (cooked)	1337	0.61	8.20	0.63
12. Fresh or dried pasta, noodles, rice and grains	192	3.41	6.50	0.50
9gii. Soups (dry soup only; concentrated)	4624	0.13	6.20	0.48
18g. Other Asian-style sauces	1914	0.26	5.00	0.39
14b. Processed fish and seafood products, raw	417	0.70	2.90	0.22
2b. Cakes and sponges	180	1.06	1.90	0.15
4a. Solids and powders	145	1.30	1.90	0.14
3b. Nuts, seeds and kernels	298	0.53	1.60	0.12
3d. Extruded snacks	570	0.24	1.40	0.10
2c. Pies and pastries	132	0.82	1.10	0.08
19b. Processed eggs and egg products	816	0.10	0.80	0.06
14c. Processed fish and seafood products, non-heat-treated	1710	0.04	0.80	0.06
3c. Potato, vegetable and grain chips	593	0.12	0.70	0.05
2a. Cookies and sweet biscuits	230	0.19	0.40	0.03
2d. Baked and cooked desserts	68	0.40	0.30	0.02
6b. Highly processed breakfast cereals	100	0.27	0.30	0.02
6a. Minimally processed breakfast cereals (includes all types – prepared, ready-made and dry mixes)	20	1.20	0.20	0.02
16c. Olives and sundried tomatoes	91	0.23	0.20	0.02
9d. Sandwiches and wraps	388	0.05	0.20	0.02
9bi. Pasta, noodles and rice or grains with sauce or seasoned (prepared)	37	0.37	0.10	0.01
2e. Pancakes, waffles and French toast	292	0.04	0.10	0.01
14a. Canned fish	660	0.02	0.10	0.01
8c. Semi-hard ripened cheese	600	0.01	0.10	0.01
1b. Nut butters	334	0.02	0.10	0.01
14d. Raw meat products and preparations	536	0.01	0.10	0.00
18d. Emulsion-based dips, sauces and dressings	760	0.00	0.00	0.00
18aii. Bouillon and soup stock (concentrated)	5682	0.00	0.00	0.00
18c. Dips and dipping sauces	23	0.07	0.00	0.00
1a. Granola and cereal-type bars	180	0.00	0.00	0.00
**Total**	**NA**	**NA**	**1302.5**	**100.00**

NA: not applicable.

^a^ In 2021, the China Health and Nutrition Survey recorded no consumption for 12 of the 55 subcategories listed in Table 1.

^b^ Subcategories are ordered from high to low according to their contribution to population sodium intake.

^c^ Food subcategories were derived from the World Health Organization’s food categorization framework,^13^ with some adaptation to take China-specific food products into account.

^d^ The sodium content of food products was derived from the FoodSwitch database.^31^

^e^ Food consumption data were obtained from the China Health and Nutrition Survey for 2018.^32^

^f^ Population sodium intake from foods in a subcategory was calculated as the median sodium content of foods in the subcategory multiplied by their consumption and divided by 100.

### Different types of targets

Table 3 shows the maximum sodium content levels, the proportion of food products to be reformulated, and the estimated sodium content reduction in food subcategories when different types of maximum sodium content target were adopted. To achieve the 90th percentile, 75th percentile, 20% reduction and WHO benchmark targets, 10.0%, 25.0%, 25.0% and 46.9%, respectively, of all food products would have to be reformulated. As a result, sodium intake from pre-packaged foods would fall from a baseline mean of 1514.0 mg/day per person by 96.2 mg/day per person (6.4%; 90th percentile target), 262.0 mg/day per person (17.3%; 75th percentile target), 302.8 mg/day per person (20.0%; 20% reduction) and 701.2 mg/day per person (46.3%; WHO benchmark). The change in mean provides a good estimate of the effect of the targets, because reformulation impacts only products with a particularly high sodium content and, consequently, the median may be little changed. The corresponding reductions in mean population sodium intake with the four targets would be 1.9% (90th percentile), 5.2% (75th percentile), 6.0% (20% reduction) and 13.9% (WHO benchmark), given that 30.1% of population sodium intake comes from pre-packaged foods.

**Table 3:** Maximum sodium content of food products, reduction in mean sodium content and proportion of products to be reformulated, by sodium reduction target and food product subcategory, China, 2021

Food product subcategory classification and description^a^ (*n* = 55)	Parameters for food products in subcategory with target achieved												
	90th percentile target^b^			75th percentile target^c^			20% reduction target				WHO benchmarks		
	Maximum sodium content, mg/100 g	Estimated decrease in mean sodium content, mg/100 g (%)		Maximum sodium content, mg/100 g	Estimated decrease in mean sodium content, mg/100 g (%)		Maximum sodium content, mg/100 g	Proportion of food products to be reformulated,%	Estimated decrease in mean sodium content, mg/100 g (%)		Maximum sodium content, mg/100 g	Proportion of food products to be reformulated, %	Estimated decrease in mean sodium content, mg/100 g (%)
1a. Granola and cereal-type bars	354	14.0 (7.0)		276	26.1 (13.0)		227	31.4	40.3 (20.0)		NA^d^	0.0	0.0 (0.0)
1b. Nut butters	520	0.3 (0.1)		488	7.0 (2.2)		325	52.2	62.4 (20.0)		NA^d^	0.0	0.0 (0.0)
2a. Cookies and sweet biscuits	480	15.4 (5.9)		347	36.8 (14.1)		295	33.9	52.1 (20.0)		265	40.3	63.3 (24.3)
2b. Cakes and sponges	360	16.6 (8.3)		271	31.2 (15.5)		239	31.8	40.1 (20.0)		205	40.1	52.5 (26.1)
2c. Pies and pastries	386	33.7 (17.4)		235	58.6 (30.2)		344	13.2	38.7 (20.0)		120	53.6	102.9 (53.1)
2d. Baked and cooked desserts	260	27.4 (22.8)		110	50.8 (42.2)		298	7.6	24.0 (20.0)		100	30.4	53.8 (44.7)
2e. Pancakes, waffles and French toast	412	13.2 (4.5)		363	22.7 (7.8)		272	54.4	58.3 (20.0)		330	35.3	32.1 (11.0)
2g. Dry mixes for making cakes, sweet biscuits, pastries and other sweet bakery wares	437	23.4 (8.7)		305	44.1 (16.4)		280	41.9	53.5 (20.0)		NA^d^	0.0	0.0 (0.0)
3a. Crackers and savoury biscuits	806	23.5 (4.8)		634	51.8 (10.6)		500	40.8	98.1 (20.0)		600	30.5	61.2 (12.5)
3b. Nuts, seeds and kernels	865	63.7 (15.4)		574	111.8 (27.0)		719	16.1	82.7 (20.0)		280	51.8	221.3 (53.5)
3c. Potato, vegetable and grain chips	1 336	53.4 (7.7)		838	128.0 (18.6)		799	27.5	138.1 (20.0)		500	62.9	265.9 (38.5)
3d. Extruded snacks	1 835	84.2 (10.9)		874	222.1 (28.7)		1239	14.0	154.5 (20.0)		520	54.4	359.3 (46.5)
4a. Solids and powders	400	60.1 (26.6)		250	85.9 (38.1)		706	2.6	45.2 (20.0)		NA^d^	0.0	0.0 (0.0)
6a. Minimally processed breakfast cereals (includes all types – prepared, ready-made and dry mixes)	260	16.0 (17.3)		146	33.9 (36.5)		238	11.7	18.5 (20.0)		100	29.3	46.7 (50.4)
6b. Highly processed breakfast cereals	379	34.3 (19.6)		236	57.8 (32.9)		371	10.1	35.1 (20.0)		280	19.7	47.7 (27.2)
8a. Fresh unripened cheese	1 563	45.9 (6.4)		953	139.5 (19.3)		936	28.6	144.2 (20.0)		190	92.9	536.3 (74.4)
8c. Semi-hard ripened cheese	1 260	52.6 (8.3)		745	121.7 (19.1)		723	26.0	127.4 (20.0)		625	45.9	164.5 (25.9)
8g. Processed cheese	1 090	36.3 (6.2)		900	65.3 (11.2)		729	34.9	116.8 (20.0)		720	34.9	119.9 (20.6)
9bi. Pasta, noodles and rice or grains with sauce or seasoned (prepared)	446	79.8 (41.4)		85	146.7 (76.1)		929	8.4	38.6 (20.0)		230	19.8	114.4 (59.3)
9bii. Pasta, noodles and rice or grains with sauce or seasoned (dry mix, concentrated)	2 680	305.4 (18.6)		2130	396.7 (24.1)		2481	13.8	329.1 (20.0)		770	67.4	1032.3 (62.7)
9c. Pizza and pizza snacks	684	32.2 (6.8)		544	53.4 (11.3)		435	46.6	94.3 (20.0)		450	44.8	87.3 (18.5)
9d. Sandwiches and wraps	925	10.4 (2.3)		616	60.6 (13.3)		519	38.0	91.3 (20.0)		430	43.0	127.2 (27.8)
9e. Prepared salads	221	6.3 (4.1)		174	15.8 (10.2)		138	60.0	30.6 (20.0)		390	0.0	0.0 (0.0)
9f. Ready-to-eat meals composed of a combination of carbohydrate and either vegetable or meat, or all three combined	2 657	27.8 (3.2)		1232	306.1 (35.1)		1796	21.2	174.7 (20.0)		250	69.1	679.1 (77.8)
9gi. Soups (ready-to-serve, canned and refrigerated soups)	1 234	35.1 (6.4)		507	180.6 (32.7)		831	21.4	110.5 (20.0)		235	92.9	318.2 (57.5)
9gii. Soups (dry soup only; concentrated)	11 500	281.2 (5.3)		8330	775.5 (14.7)		7330	30.3	1052.2 (20.0)		1200	76.5	4238.5 (80.6)
10a. Salted butter, butter blends, margarine and oil-based spreads	800	4.8 (1.2)		695	26.8 (6.6)		533	47.5	81.1 (20.0)		400	59.3	152.7 (37.7)
11a. Sweet and raisin breads	360	10.7 (4.5)		277	24.0 (10.0)		215	54.7	48.0 (20.0)		310	17.4	17.1 (7.1)
11b. Leavened bread	433	17.2 (6.1)		361	28.8 (10.2)		280	42.7	56.6 (20.0)		330	31.9	37.9 (13.4)
11c. Flatbreads	496	29.1 (9.1)		411	43.4 (13.5)		347	41.0	64.4 (20.0)		320	47.9	76.2 (23.7)
12. Fresh or dried pasta, noodles, rice and grains	1 030	51.4 (12.9)		650	106.4 (26.7)		796	16.1	79.5 (20.0)		NA^d^	0.0	0.0 (0.0)
14a. Canned fish	1 400	62.3 (7.9)		977	132.3 (16.7)		886	32.3	158.0 (20.0)		360	85.6	447.9 (56.6)
14b. Processed fish and seafood products, raw	1 380	267.4 (34.1)		690	370.4 (47.3)		3339	3.8	156.6 (20.0)		270	68.2	556.1 (71.0)
14c. Processed fish and seafood products, non-heat-treated	5 063	18.4 (0.7)		4892	58.0 (2.2)		3302	32.9	516.1 (20.0)		800	90.8	1813.2 (70.3)
14d. Raw meat products and preparations	1 007	110.1 (17.2)		735	154.7 (24.2)		869	15.6	127.9 (20.0)		230	79.0	436.6 (68.3)
14ei. Whole muscle meat products, heat-treated (frozen and canned products)	1 400	46.9 (5.7)		932	124.0 (15.2)		809	40.5	163.7 (20.0)		270	89.4	561.8 (68.7)
14eii. Whole muscle meat products, heat-treated (refrigerated products)	1 580	50.9 (5.3)		1370	90.1 (9.4)		1090	40.0	192.5 (20.0)		600	66.2	452.4 (47.0)
14f. Whole muscle meat products, non-heat preservation	1 934	39.3 (2.8)		1708	79.4 (5.6)		1259	63.0	282.2 (20.0)		950	81.5	505 (35.8)
14g. Comminuted meat products, heat-treated (cooked)	1 740	47.6 (4.2)		1349	112.7 (10.0)		1034	50.3	226.3 (20.0)		540	93.0	603.6 (53.4)
14h. Comminuted meat products, non-heat preservation	2 654	70.3 (4.5)		2211	155.4 (9.9)		1734	46.8	313.0 (20.0)		830	73.4	816.1 (52.1)
14i. Processed fish and seafood, heat-treated (cooked)	2 160	114.5 (8.1)		1694	190.8 (13.5)		1425	44.1	282.6 (20.0)		NA^d^	0.0	0.0 (0.0)
16b. Pickled vegetables	3 069	205.9 (10.8)		2292	329.2 (17.3)		2110	32.2	380.7 (20.0)		550	88.2	1387.3 (72.9)
16c. Olives and sundried tomatoes	1 675	310.5 (43.5)		519	506.4 (71.0)		4745	3.8	142.8 (20.0)		780	20.2	447.9 (62.8)
16g. Battered or breaded vegetables	898	243.0 (41.2)		570	288.0 (48.8)		2928	2.3	118.0 (20.0)		510	27.9	304.1 (51.6)
17a. Tofu and tempeh	1 568	52.0 (5.6)		1212	113.3 (12.1)		992	43.7	187.0 (20.0)		280	84.5	689.0 (73.6)
17b. Meat analogues	2 696	130.2 (7.6)		2413	174.4 (10.2)		1906	40.9	341.2 (20.0)		250	97.7	1458.6 (85.5)
18aii. Bouillon and soup stock (concentrated)	14 576	875.5 (12.3)		8327	1819.0 (25.5)		10 324	16.1	1425.6 (20.0)		15 000	10.1	832.5 (11.7)
18b. Cooking sauces including pasta sauces and tomato sauces (not concentrated)	3 364	238.9 (20.8)		981	590.3 (51.3)		3456	9.1	230.2 (20.0)		330	74.9	874.0 (75.9)
18c. Dips and dipping sauces	700	224.2 (65.9)		62	308.0 (90.6)		2672	5.4	68.0 (20.0)		360	12.1	262.0 (77.1)
18d. Emulsion-based dips, sauces and dressings	1 573	77.9 (8.2)		1196	133.2 (14)		1014	35.7	189.9 (20.0)		500	89.3	480.8 (50.6)
18e. Condiments	4 972	211.0 (7.4)		3780	404.9 (14.2)		3204	34.9	571.0 (20.0)		650	91.7	2229.0 (78.1)
18f. Soy sauce and fish sauce	7 900	170.7 (3.3)		6830	357.6 (6.9)		5059	52.5	1042.6 (20.0)		4840	55.2	1160.9 (22.3)
18g. Other Asian-style sauces	4 207	189.4 (8.4)		3758	287.3 (12.7)		3199	31.8	452.4 (20.0)		680	72.7	1692.3 (74.8)
18h. Marinades and thick pastes	9 550	822.1 (20.0)		5118	1532.3 (37.2)		9541	10.2	823.0 (20.0)		1425	63.6	3083.3 (74.9)
19b. Processed eggs and egg products	2 100	115.7 (10.0)		1396	250.5 (21.6)		1473	23.2	232.0 (20.0)		NA^d^	0.0	0.0 (0.0)
**Overall**	NA	96.2 (6.4)		NA	262.0 (17.3)		NA	25.0	302.8 (20.0)		NA	46.9	701.2 (46.3)

NA: not applicable; WHO: World Health Organization.

^a^ Food subcategories were based on the World Health Organization’s food categorization framework,^13^ with some adaptation to take China-specific food products into account.

^b^ With the 90th percentile target, 10% of products in each category had to be reformulated.

^c^ With the 75th percentile target, 25% of products in each category had to be reformulated.

^d^ As WHO did not set benchmarks for this subcategory, we assumed no foods had to be reformulated.^13^

**Fig. 2:**
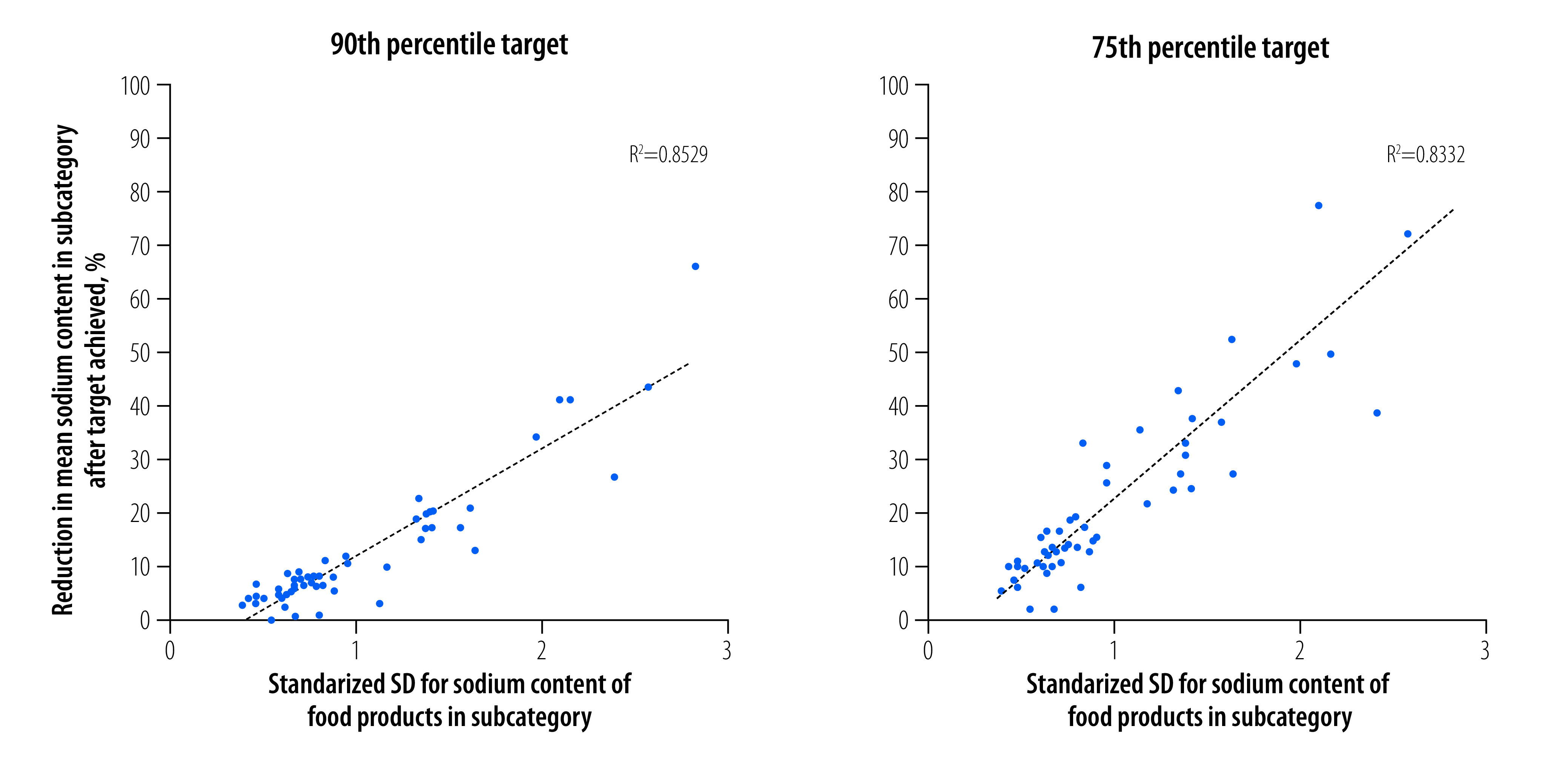
Relationship between the variation in sodium content of food products in a subcategory and the reduction in sodium content with different sodium content targets, China, 2021

**Fig. 3:**
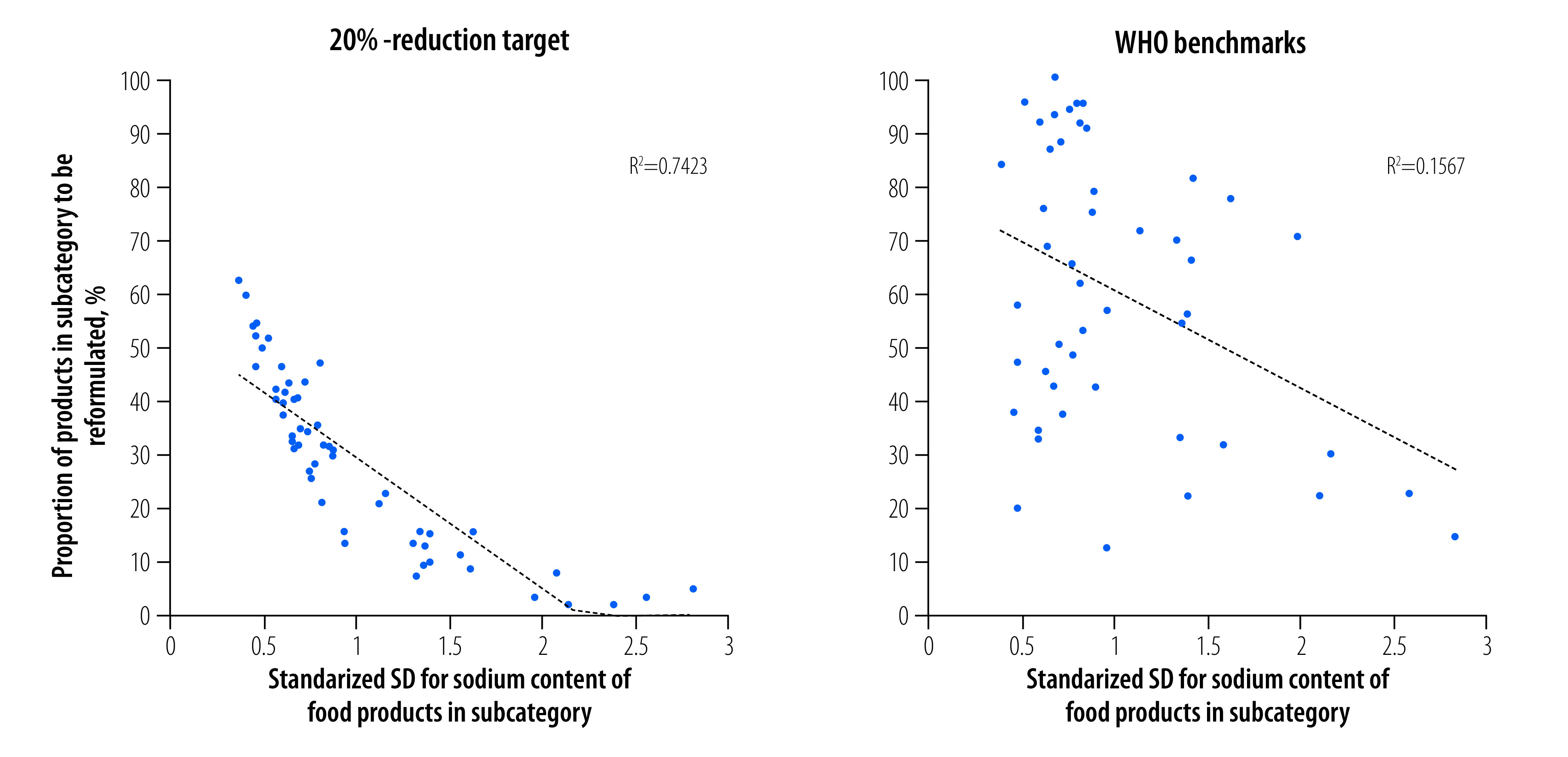
Relationship between the variation in sodium content of food products in a subcategory and the proportion of products to be reformulated with different sodium content targets, China, 2021

SD: standard deviation; WHO: World Health Organization.

Note: *R^2^* represents the proportion of the variance in the dependent variable (i.e. the proportion of products to be reformulated in the subcategory) that is explained by the independent variable (i.e. the standardized standard deviation of the sodium content of food products in the subcategory) in a linear regression model. Food subcategories were derived from the World Health Organization’s food categorization framework,^13^ with some adaptation to take China-specific food products into account.

In the analysis, we found that the reduction in mean sodium content with percentile targets varied greatly across subcategories and was positively correlated with the standardized SD for sodium content in the subcategories (Fig. 2). In addition, with the 20% reduction target, the proportion of food products that had to be reformulated in a subcategory correlated negatively with the standardized SD (Fig. 3). In contrast, with the WHO benchmarks, the proportion to be reformulated had no clear association with the standardized SD (Fig. 3). On examining the data in Table 1, Table 2, Table 3, Fig. 2 and Fig. 3 together, we found that several major sodium contributors, such as soy sauce and fish sauce (subcategory 18f) and whole-muscle meat products with non-heat preservation (subcategory 14f), had relatively high and stable sodium contents (i.e. a standardized SD less than 0.5). Although a greater number of products in these two subcategories should be reformulated, the 75th percentile target, for example, would lead to only a small reduction in sodium content (e.g. 6.9% for subcategory 18f and 5.6% for subcategory 14f; Table 3). In contrast, with the 20% reduction target, a fixed reduction in sodium content of 20% would be guaranteed for all subcategories; however, in subcategories with a large, standardized SD, too few products would have to be reformulated. 

SD: standard deviation.

Note: *R^2^* represents the proportion of the variance in the dependent variable (i.e. the reduction in mean sodium content of foods in the subcategory after the target has been achieved) that is explained by the independent variable (i.e. the standardized standard deviation of the sodium content of food products in the subcategory) in a linear regression model. Food subcategories were derived from the World Health Organization’s food categorization framework,^13^ with some adaptation to take China-specific food products into account.

### Sodium content targets

Among the different types of sodium content target we investigated, both the 75th percentile and the 20% reduction targets would reduce mean sodium intake from packaged foods by about 20%: the reductions would be 17.3% and 20%, respectively. After taking into account our criteria for selecting suitable targets and the results of our analysis, our initial proposal was that the 20% reduction target should be used for 47 food subcategories, and the 90th percentile target should be used for eight (Table 4). The 90th percentile target was used for the eight subcategories in which fewer than 10% of products would have had to be reformulated if the 20% reduction target were adopted. This proposal could lead to a reduction in sodium intake from pre-packaged foods of 305.0 mg/day per person, which represents a reduction of 20.1% from a baseline mean of 1514 mg/day per person, and a 6.1% reduction in population sodium intake in China. (Table 4).

**Table 4:** Proposed maximum sodium content targets for food product subcategories and their effect on population sodium intake, China, 2021

Food product subcategory classification and description^a,b^ (*n* = 55)	Parameters for food products in subcategory						
	Maximum sodium content target,^c^ mg/100 g	Mean sodium content before reformulation, mg/100 g	% of products to be reformulated	Reduction in mean sodium content after reformulation, mg/100 g (%)	Reduction in mean population sodium intake after reformulation,^d^ mg/day per person	Proportional contribution to sodium reduction among all pre-packaged foods, %	Cumulative contribution to sodium reduction among all pre-packaged foods, %
18f. Soy sauce and fish sauce	5 059	5213	52.5	1043 (20.0)	123.8	40.6	40.6
9f. Ready-to-eat meals composed of a combination of carbohydrate and either vegetable or meat, or all three combined	1 796	873	21.2	175 (20.0)	54.3	17.8	58.4
9bii. Pasta, noodles and rice or grains with sauce or seasoned (dry mix, concentrated)	2 481	1646	13.8	329 (20.0)	21.1	6.9	65.3
16b. Pickled vegetables	2 110	1903	32.2	381 (20.0)	18.3	6.0	71.3
10a. Salted butter, butter blends, margarine and oil-based spreads	533	405	47.5	81 (20.0)	15.5	5.1	76.4
18b. Cooking sauces including pasta sauces and tomato sauces (not concentrated)	3 364^e^	1151	10.0	239 (20.8)	13.2	4.3	80.7
14f. Whole muscle meat products, non-heat preservation	1 259	1411	63.0	282 (20.0)	12.1	4.0	84.7
17a. Tofu and tempeh	992	936	43.7	187 (20.0)	8.2	2.7	87.4
18h. Marinades and thick pastes	9 541	4115	10.2	823 (20.0)	5.8	1.9	89.3
18e. Condiments	3 204	2855	34.9	571 (20.0)	5.1	1.7	91.0
14g. Comminuted meat products, heat-treated (cooked)	1 034	1131	50.3	226 (20.0)	4.8	1.6	92.5
14h. Comminuted meat products, non-heat preservation	1 734	1566	46.8	313 (20.0)	4.3	1.4	94.0
11b. Leavened bread	280	282	42.7	57 (20.1)	2.8	0.9	94.9
12. Fresh or dried pasta, noodles, rice and grains	796	398	16.1	80 (20.0)	2.7	0.9	95.8
3a. Crackers and savoury biscuits	500	490	40.8	98 (20.0)	2.1	0.7	96.4
14b. Processed fish and seafood products, raw	1 380^e^	783	9.2	267 (34.2)	1.9	0.6	97.0
14i. Processed fish and seafood, heat-treated (cooked)	1 425	1413	44.1	283 (20.0)	1.7	0.6	97.6
9gii. Soups (dry soup only; concentrated)	7 330	5261	30.3	1052 (20.0)	1.4	0.4	98.1
18g. Other Asian-style sauces	3 199	2262	31.8	452 (20.0)	1.2	0.4	98.4
4a. Solids and powders	400^e^	226	9.7	60 (26.6)	0.8	0.3	98.7
16c. Olives and sundried tomatoes	1 675^e^	714	10.0	310 (43.5)	0.7	0.2	98.9
3b. Nuts, seeds and kernels	719	414	16.1	83 (20.0)	0.4	0.1	99.1
2b. Cakes and sponges	239	201	31.8	40 (20.0)	0.4	0.1	99.2
3d. Extruded snacks	1 239	773	14.0	154 (20.0)	0.4	0.1	99.3
2c. Pies and pastries	344	194	13.2	39 (20.0)	0.3	0.1	99.4
9bi. Pasta, noodles and rice or grains with sauce or seasoned (prepared)	446^e^	193	10.2	80 (41.4)	0.3	0.1	99.5
19b. Processed eggs and egg products	1 473	1160	23.2	232 (20.0)	0.2	0.1	99.6
6a. Minimally processed breakfast cereals (includes all types – prepared, ready-made and dry mixes)	238	93	11.7	18 (19.9)	0.2	0.1	99.7
14c. Processed fish and seafood products, non-heat-treated	3 302	2581	32.9	516 (20.0)	0.2	0.1	99.7
3c. Potato, vegetable and grain chips	799	690	27.5	138 (20.0)	0.2	0.1	99.8
18c. Dips and dipping sauces	700^e^	340	9.7	224 (65.9)	0.2	0.1	99.9
2d. Baked and cooked desserts	260^e^	120	10.9	27 (22.8)	0.1	0.0	99.9
2a. Cookies and sweet biscuits	295	261	33.9	52 (20.0)	0.1	0.0	99.9
6b. Highly processed breakfast cereals	371	175	10.1	35 (20.0)	0.1	0.0	100.0
9d. Sandwiches and wraps	519	457	38.0	91 (20.0)	0.0	0.0	100.0
14a. Canned fish	886	791	32.3	158 (20.0)	0.0	0.0	100.0
2e. Pancakes, waffles and French toast	272	292	54.4	58 (19.9)	0.0	0.0	100.0
14d. Raw meat products and preparations	869	639	15.6	128 (20.0)	0.0	0.0	100.0
8c. Semi-hard ripened cheese	723	636	26.0	127 (20.0)	0.0	0.0	100.0
1b. Nut butters	325	313	52.2	62 (19.9)	0.0	0.0	100.0
1a. Granola and cereal-type bars	227	201	31.4	40 (20.0)	0.0	0.0	100.0
2g. Dry mixes for making cakes, sweet biscuits, pastries and other sweet bakery wares	280	268	41.9	54 (19.9)	0.0	0.0	100.0
8a. Fresh unripened cheese	936	721	28.6	144 (20.0)	0.0	0.0	100.0
8g. Processed cheese	729	583	34.9	117 (20.0)	0.0	0.0	100.0
9c. Pizza and pizza snacks	435	471	46.6	94 (20.0)	0.0	0.0	100.0
9e. Prepared salads	138	154	60.0	31 (19.8)	0.0	0.0	100.0
9gi. Soups (ready-to-serve, canned and refrigerated soups)	831	553	21.4	110 (20.0)	0.0	0.0	100.0
11a. Sweet and raisin breads	215	240	54.7	48 (20.0)	0.0	0.0	100.0
11c. Flatbreads	347	321	41.0	64 (20.1)	0.0	0.0	100.0
14ei. Whole muscle meat products, heat-treated (frozen and canned products)	809	818	40.5	164 (20.0)	0.0	0.0	100.0
14eii. Whole muscle meat products, heat-treated (refrigerated products)	1 090	962	40.0	193 (20.0)	0.0	0.0	100.0
16g. Battered or breaded vegetables	898^e^	590	11.6	243 (41.2)	0.0	0.0	100.0
17b. Meat analogues	1 906	1706	40.9	341 (20.0)	0.0	0.0	100.0
18aii. Bouillon and soup stock (concentrated)	10 324	7129	16.1	1426 (20.0)	0.0	0.0	100.0
18d. Emulsion-based dips, sauces and dressings	1 014	950	35.7	190 (20.0)	0.0	0.0	100.0
**Total**	NA	NA	26.0^f^	NA	305.0	100.0	100.0

NA: not applicable.

^a^ Subcategories are ordered from high to low according to their proportional contribution to the reduction in population sodium intake on achievement of the targets.

^b^ Food subcategories were derived from the World Health Organization’s food categorization framework,^13^ with some adaptation to take China-specific food products into account.

^c^ Targets were based on the 20% reduction target, except where indicated by the superscript letter e.

^d^ For each subcategory, the reduction in population sodium intake was calculated as the reduction in mean sodium content (mg/100 g) multiplied by consumption (g/day per person; Table 2) and divided by100.

^e^ The maximum sodium content target for this subcategory was based on the 90th-percentile target.

^f^ The overall proportion of products to be reformulated across all subcategories was the sum of the number of products to be reformulated  in each subcategory divided by the total number of products.

## Discussion

Our study was triggered by the establishment of WHO’s global sodium benchmarks for different food categories, and the approach employed in developing those benchmarks. We found that pre-packaged foods accounted for a median sodium intake of 1302.5 mg/day per adult in China, which was 30.1% of population sodium intake. If all pre-packaged foods met WHO’s global sodium benchmarks, sodium intake would be 701.2 mg/day per person (46.3%) lower, equivalent to a 13.9% reduction in mean population sodium intake. After considering different types of sodium content targets for use in China, we proposed a set of revised 20% reduction targets because of their ability to induce a substantial and acceptable reduction in sodium content for most food subcategories.

In 2017, China’s National Nutrition Plan prioritized improving nutrition laws, policies and standards.^37^ As a key strategy for sodium intake reduction, setting sodium content targets for pre-packaged foods has been a hot topic, but has been held back largely by the lack of robust and reasonable sodium targets. Our proposal of revised 20% reduction targets could be a good starting point because these targets will: (i) encourage the reformulation of food products with a relatively high sodium content; (ii) guarantee a substantial reduction in sodium content (i.e. around 20% for each food subcategory), which may not be noticed by consumers and should be acceptable to most food companies;^38^^,^^39^ and (iii) help achieve the national goal of a 20% reduction in population sodium intake by 2030. Recent modelling indicates that, should the proposed targets be met and maintained until 2030, around 6 million cardiovascular disease events could be prevented.^40^ Moreover, if this approach proves successful, targets could be gradually lowered further towards WHO’s benchmarks and food reformulation could play a leading role in reducing sodium intake nationally. However, even meeting WHO’s benchmarks would reduce population sodium intake by only 13.9%. Consequently, authorities should simultaneously implement strategies targeting discretionary salt use during cooking and eating at home and in restaurants.

We found that a small number of food subcategories with a high sodium content or a large consumption, or both, accounted for almost 90% of sodium intake and could, if the targets were achieved, contribute almost 90% to the reduction in sodium intake from pre-packaged foods. There is, then, an opportunity to start target setting, voluntarily or mandatorily, with these priority foods, thereby ensuring the initial organizational, supervisory and evaluation workload is low. Other countries have considerable experience in setting targets for priority foods, especially as a starting point.^41^^–^^43^

Mandatory reformulation generally appears to achieve larger reductions in population-wide salt consumption than voluntary reformulation.^44^ If targets are implemented voluntarily, supportive measures are needed, such as strong government leadership, the threat of government regulation if compliance is low, and robust monitoring with the publication of findings.^45^

To make full use of WHO’s food categorization framework and ensure comparability between countries, we tried to minimize changes to the framework. We noticed, however, that some subcategories (e.g. various processed meat and egg product subcategories) had very similar sodium concentrations. Further consolidation of these categories might simplify the implementation of food sodium content targets.

Initially, we considered only two strategies for setting maximum targets: (i) fixed percentile targets (e.g. 50th, 75th and 90th percentiles); and (ii) existing maximum targets, such as WHO’s benchmarks. However, subsequent analysis showed that percentile targets had limitations: they resulted in only a very small reduction in the mean sodium content of food subcategories that had a small variation in sodium content between products, even though these products could have a very high sodium content and could be the main contributors to dietary sodium. For this reason, we proposed targets based on the 20% reduction strategy. We did not simulate the impact of targets included in the Guideline for Salt Reduction for the Chinese Food Industry because these targets were based on only around 9000 food products compared with our 51 803, and because they were derived by simply multiplying the highest sodium content in individual food categories by 90% or 80%.^28^ Moreover, we wanted to build on WHO’s sodium benchmarks, and the Guideline’s food categorization framework is totally different from WHO’s framework.

Our study had several limitations that could affect the extrapolation of our findings. First, we excluded some products that naturally contain sodium but no added salt, such as milk. Although this is reasonable when evaluating the effect of food reformulation on sodium intake, we may have slightly underestimated the overall contribution of pre-packaged foods to population sodium intake, and slightly overestimated the relative effect of sodium content reduction. Second, consumption-weighted sodium content reduction was based on the consumption of foods in a subcategory as a whole rather than on the consumption of individual foods. This approach may have influenced our calculations for subcategories in which the consumption of different products was uneven. Third, the consumption data set lacked participating families from west China, where the consumption of pre-packaged foods is likely to be lower than in central and east China. This exclusion may have led to the contribution of pre-packaged foods to sodium intake being overestimated. Fourth, we did not consider setting targets for priority food categories in the study, although our findings help make this possible.

In conclusion, pre-packaged food contributes to nearly one-third of population sodium intake in China and its market share is increasing. Reformulating foods is an important part of the solution. Although they will not bring sodium intake down to the level achievable with WHO’s global sodium benchmarks, our revised 20% reduction targets provide a valuable starting point for government policy. We strongly recommend that action on dietary salt in China should involve comprehensive strategies that simultaneously target both sodium in pre-packaged food and discretionary salt use.
